# Supplementary material for: Auto-encoding NMR chemical shifts from their native vector space to a residue-level biophysical index
Source: Nat Commun. 2019 Jun 7;10:2511. doi: 10.1038/s41467-019-10322-w (PMC6555786; doi:10.1038/s41467-019-10322-w)
Supplement: Supplementary file 1 — Supplementary Information [file 41467_2019_10322_MOESM1_ESM.pdf]

Supplementary Information

Auto-encoding NMR chemical shifts from their native vector space to a residue-level biophysical index

Gabriele Orlando, Daniele Raimondi, Wim Vranken

Supplementary Figures

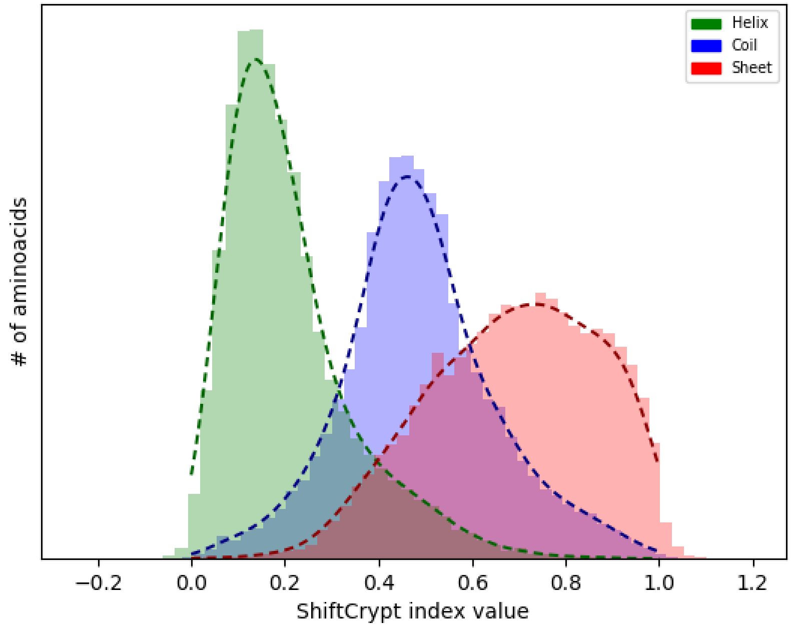

Supplementary Figure 1: **Relation between ShiftCrypt and secondary structures.** The general distribution of the ShiftCrypt index by the three secondary structure types as observed in the related experimental structures. The green, blue and red distributions represent the helix, coil and sheet distributions respectively. The distributions are normalized per secondary structure.

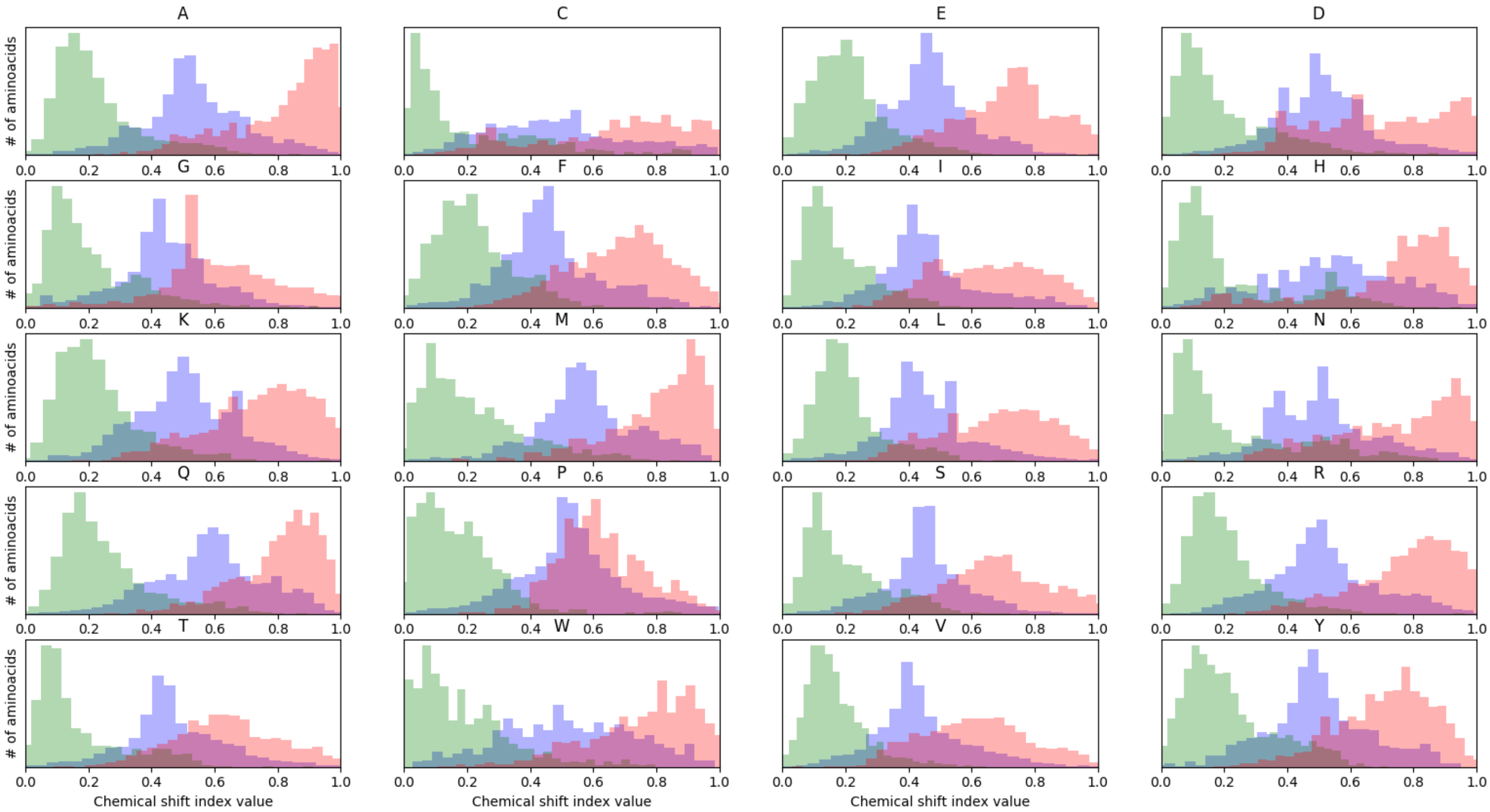

Supplementary Figure 2: **Amino acid-specific Distribution of the ShiftCrypt index.** The distribution of the ShiftCrypt values per amino acid type (each subplot) for the three secondary structure types as observed in the related experimental structure. The green, blue and red distributions represent the helix, coil and sheet distributions respectively. The distributions are normalized per secondary structure.

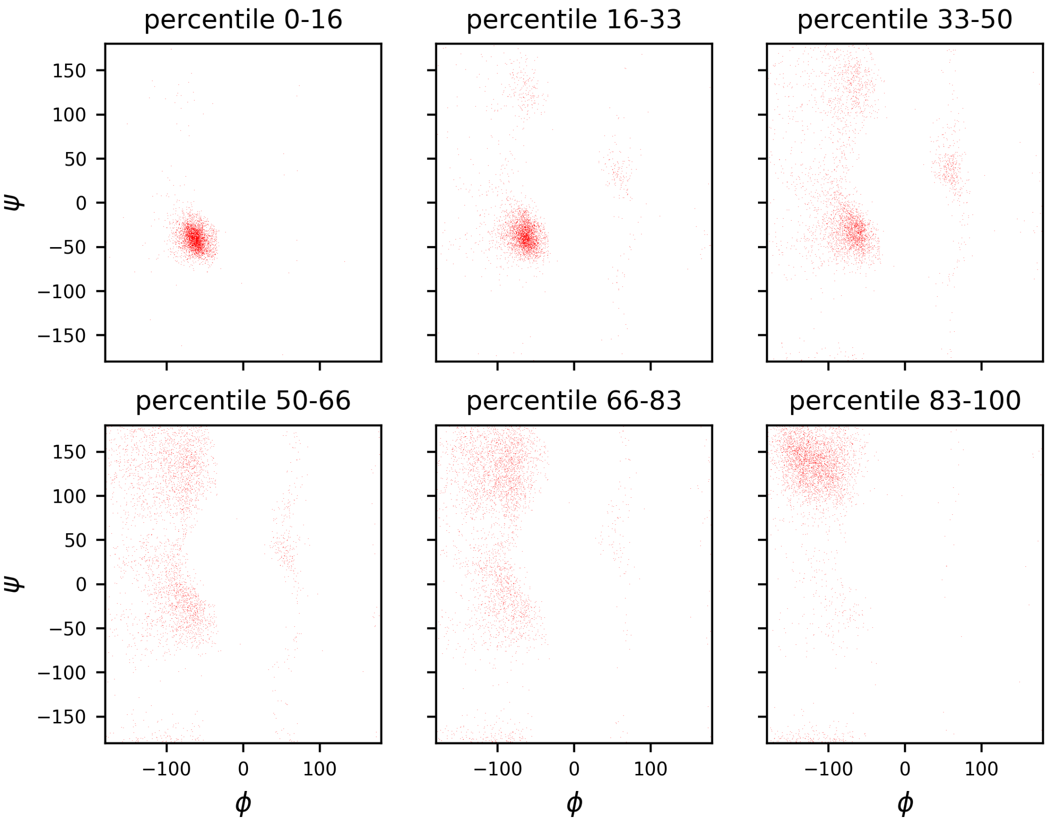

Supplementary Figure 3: **Torsion angles and ShiftCrypt for acid amino acids.** Every plot represent an interval of ShiftCrypt values. For each interval, the plot show the distribution of the Φ and Ψ angles of each residue with a ShiftCrypt index in that range

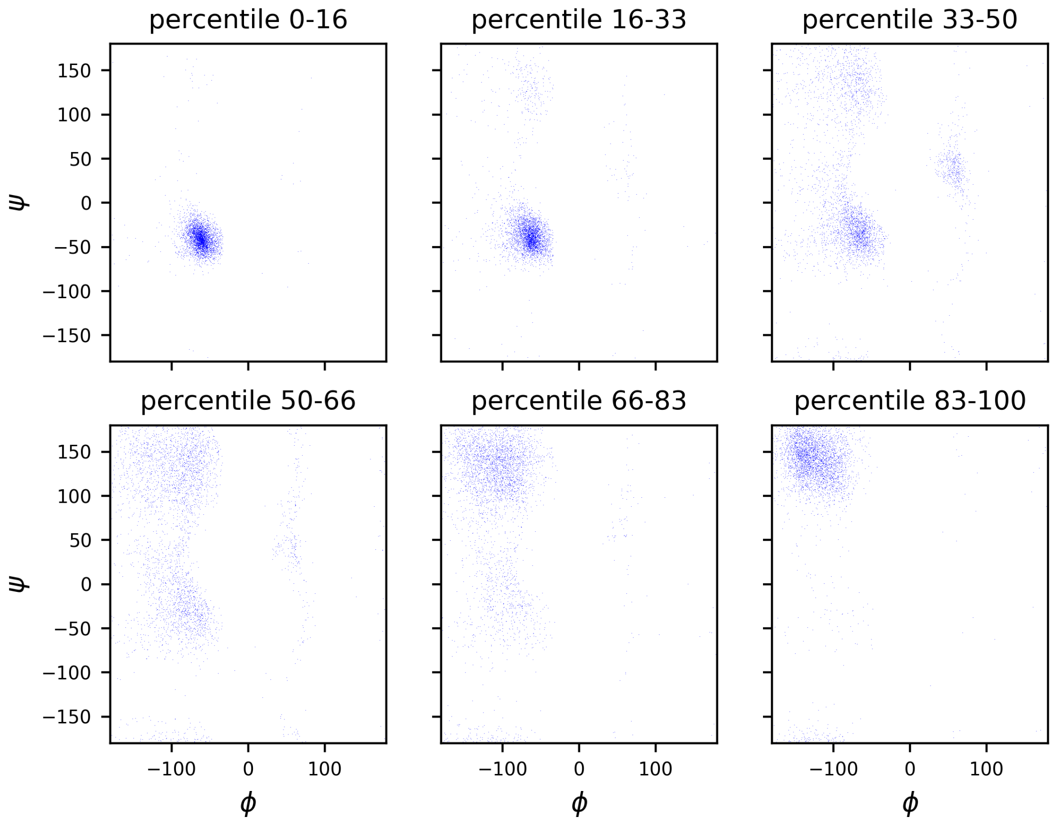

Supplementary Figure 4: **Torsion angles and ShiftCrypt for basic amino acids.** Every plot represent an interval of ShiftCrypt values. For each interval, the plot show the distribution of the Φ and Ψ angles of each residue with a ShiftCrypt index in that range

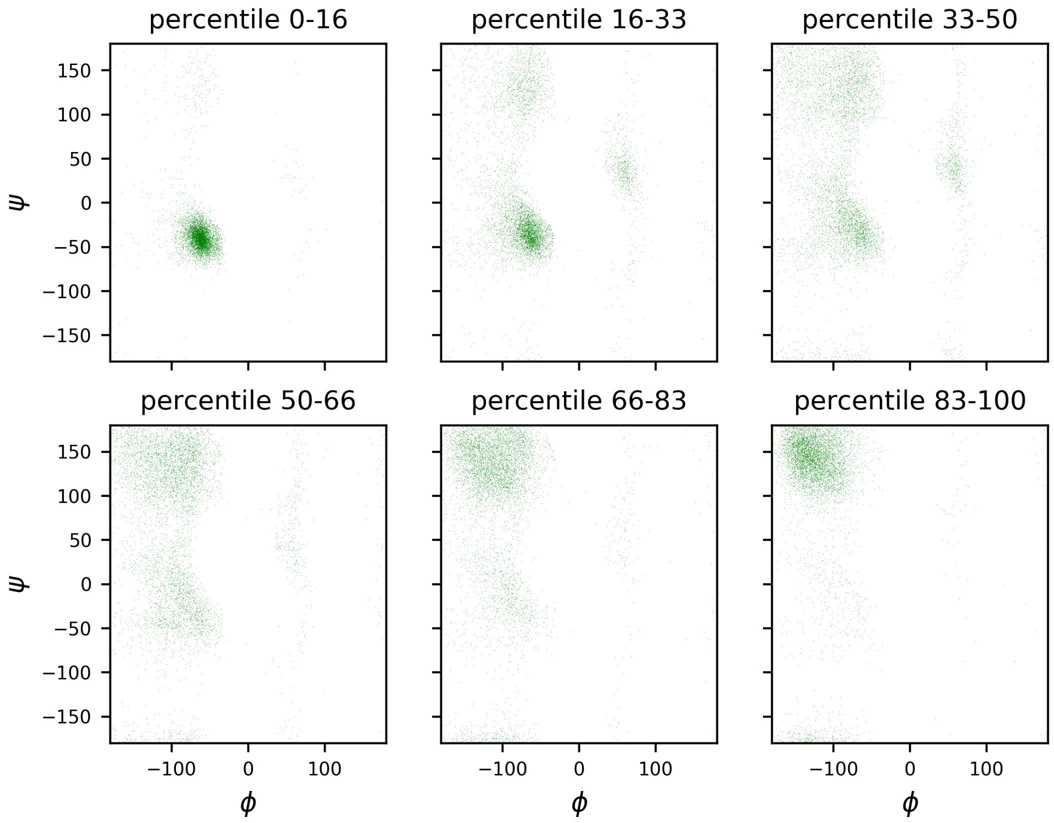

Supplementary Figure 5: **Torsion angles and ShiftCrypt for hydrophilic amino acids.** Every plot represent an interval of ShiftCrypt values. For each interval, the plot show the distribution of the Φ and Ψ angles of each residue with a ShiftCrypt index in that range

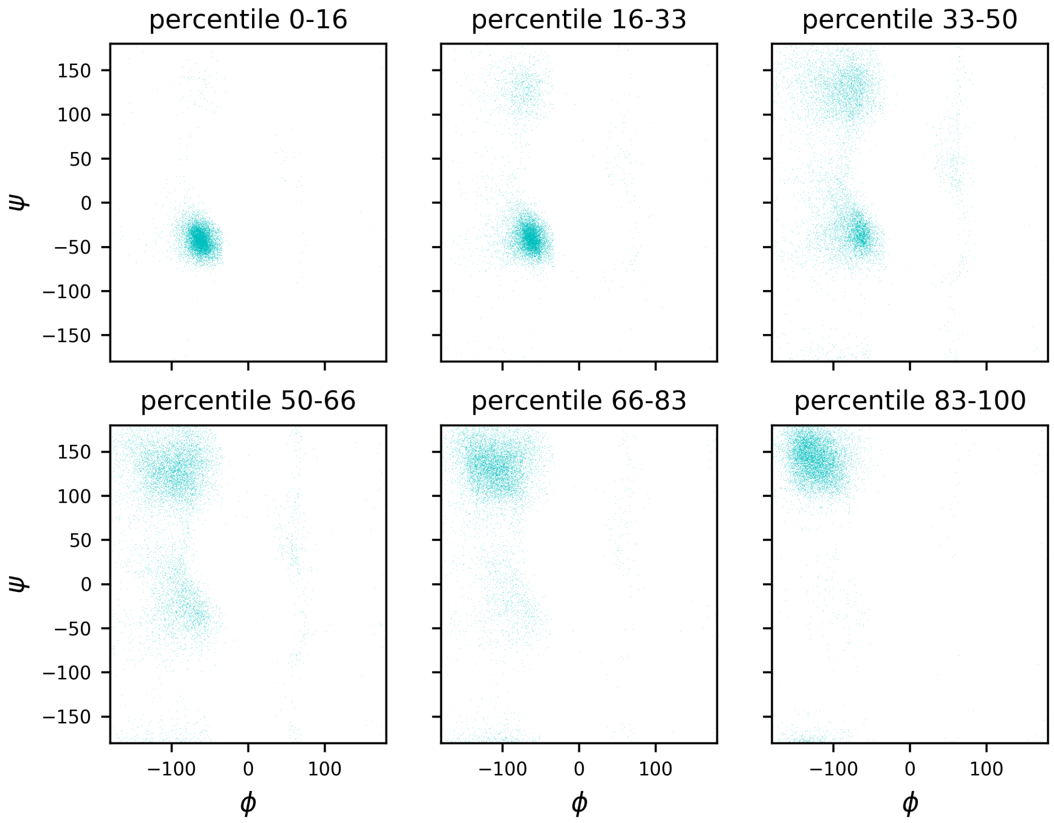

Supplementary Figure 6: **Torsion angles and ShiftCrypt for hydrophobic amino acids.** Every plot represent an interval of ShiftCrypt values. For each interval, the plot show the distribution of the Φ and Ψ angles of each residue with a ShiftCrypt index in that range

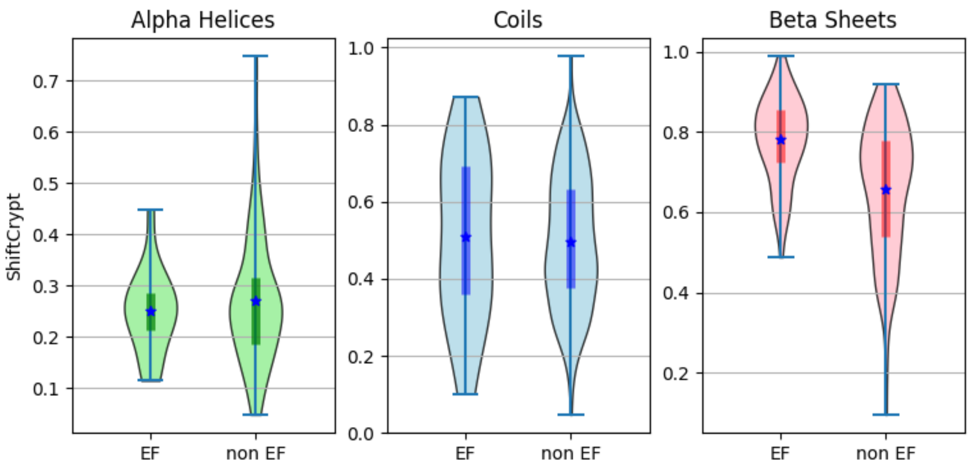

Supplementary Figure 7: **Relation between early folding residues and ShiftCrypt index.** the plot shows violin plots with encompassed boxplot showing the relation between predicted early folding residues and ShiftCrypt values. The residues are stratified by secondary structure. The blue star represent the mean of the distribution, while the region between percentiles 25 and 75 is highlighted with a darker color

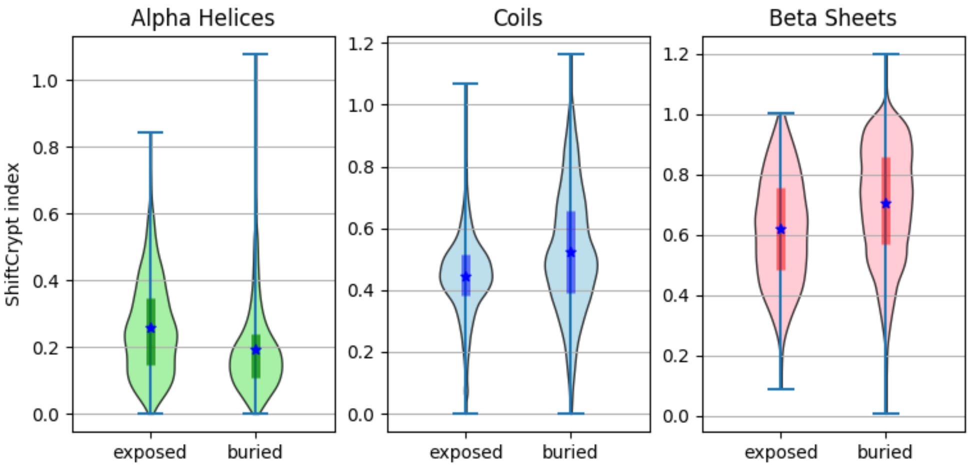

Supplementary Figure 8: **Relation between solvent accessibility and ShiftCrypt index.** The plot shows violin plots with encompassed boxplot showing the distribution of ShiftCrypt of residues classified by whether they are buried or not based on the experimental structure. We defined a residue as buried if its carbon alpha is further then 1.5 angstrom from the surface. The residues are stratified by secondary structure. The blue star represent the mean of the distribution, while the region between percentiles 25 and 75 is highlighted with a darker color

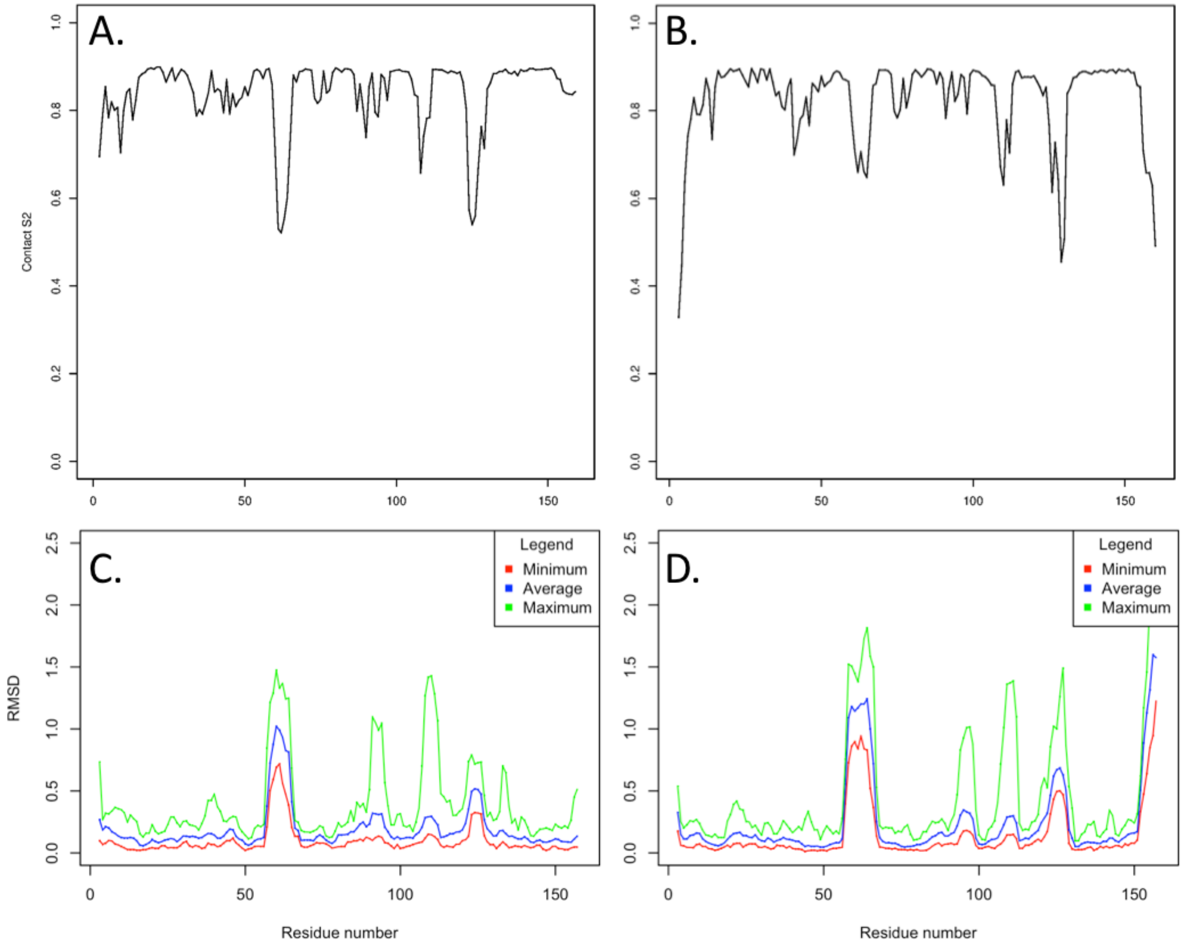

Supplementary Figure 9: **Contact S2 value and local backbone RMSD for cherry allergen.** Contact S2 value (A) and local backbone RMSD (C) for cherry allergen (PDB 1e09), and contact S2 value (B) and local backbone RMSD (D) for strawberry allergen (PDB 2lpx). The contact S2 values are calculated from all models of the NMR ensemble, the local RMSD is calculated over all pairwise pentapeptide fragments in each ensemble.

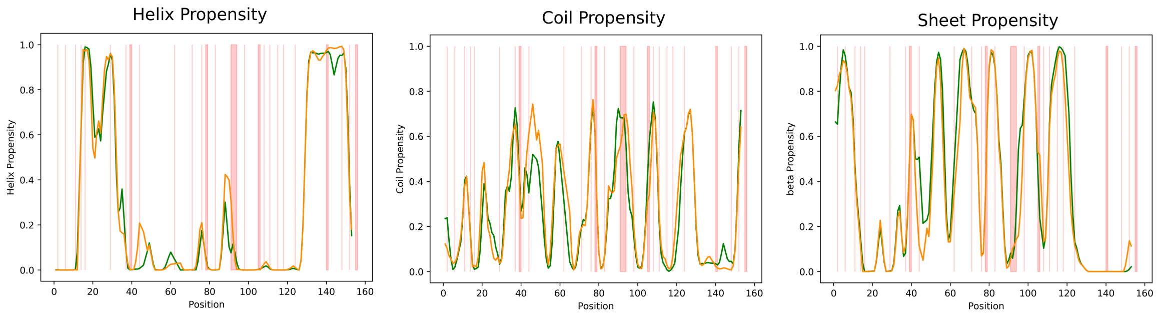

Supplementary Figure 10: **Conservation of d2D in homologous proteins.** The secondary structure propensities values determined by d2D for the strawberry (green) and cherry (orange) allergens. The red bars highlight amino acid mismatches in the alignment of the two proteins. The x-axis represents the aligned position, while the y-axis the propensity value of the two proteins in that specific residue

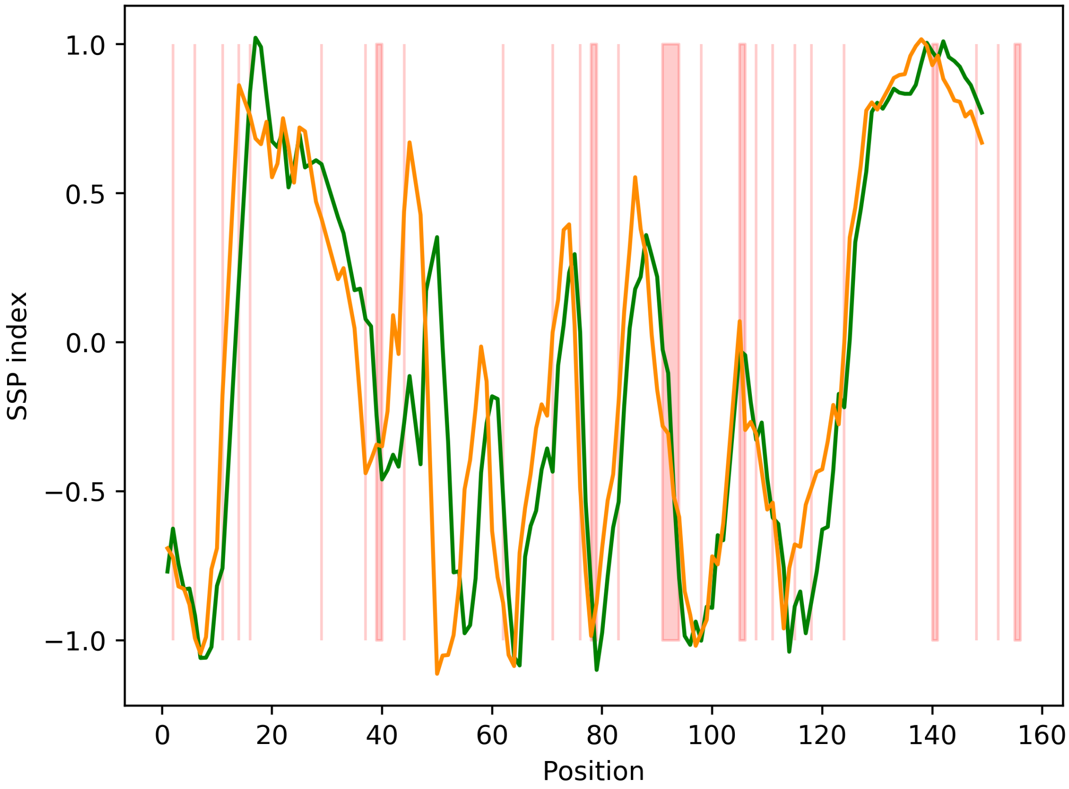

Supplementary Figure 11: **Conservation of SSP in homologous proteins.** The SSP index for the strawberry (green) and cherry (orange) allergens. The red bars highlight amino acid mismatches in the alignment of the two proteins. The x-axis represents the aligned position, while the y-axis the SSP value of the two proteins in that specific residue

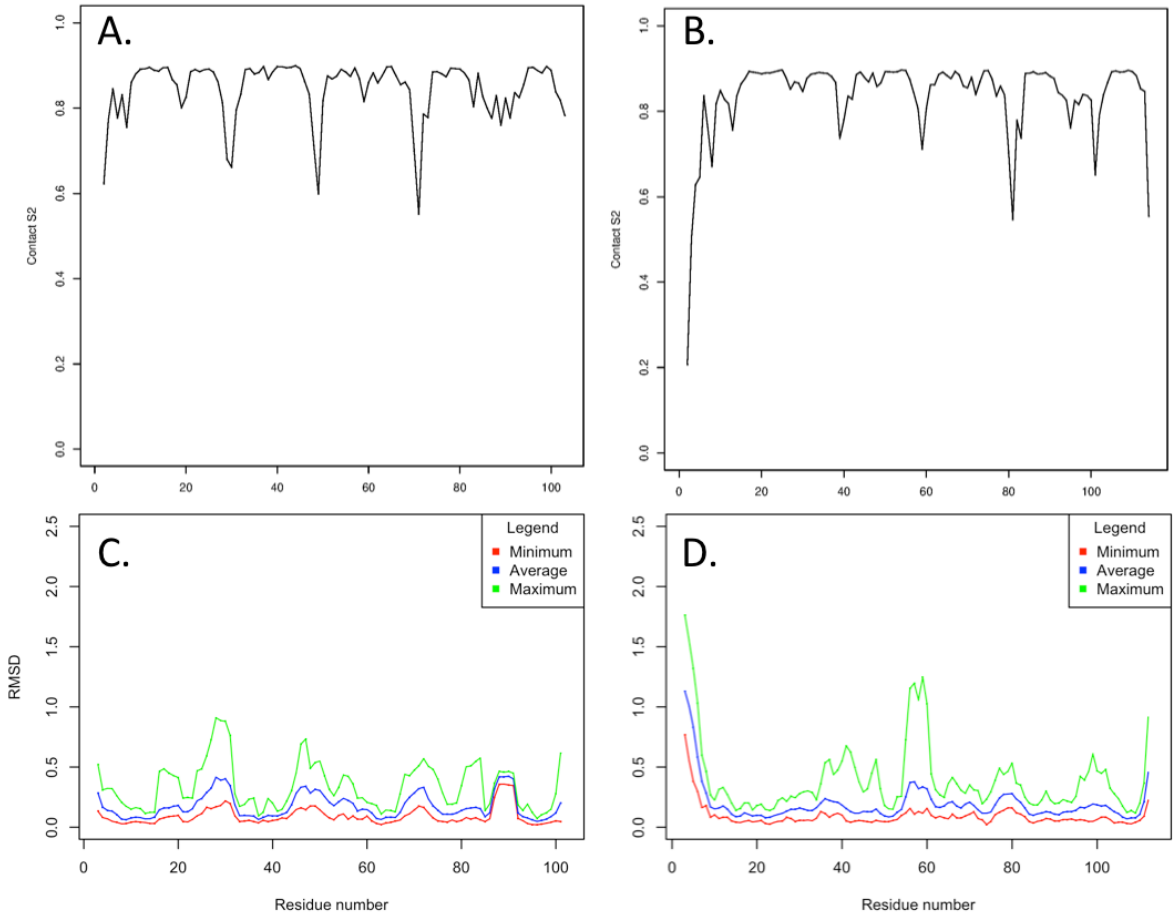

Supplementary Figure 12: **Contact S2 value and local backbone RMSD for thioredoxin-1.** Contact S2 value (A) and local backbone RMSD (C) for the oxidised thioredoxin-1 from S. cervisiae (PDB 2n5b), and contact S2 value (B) and local backbone RMSD (D) for the corresponding A. thaliana thioredoxin-1 (PDB 1xfl). The contact S2 values are calculated from all models of the NMR ensemble, the local RMSD is calculated over all pairwise pentapeptide fragments in each ensemble.

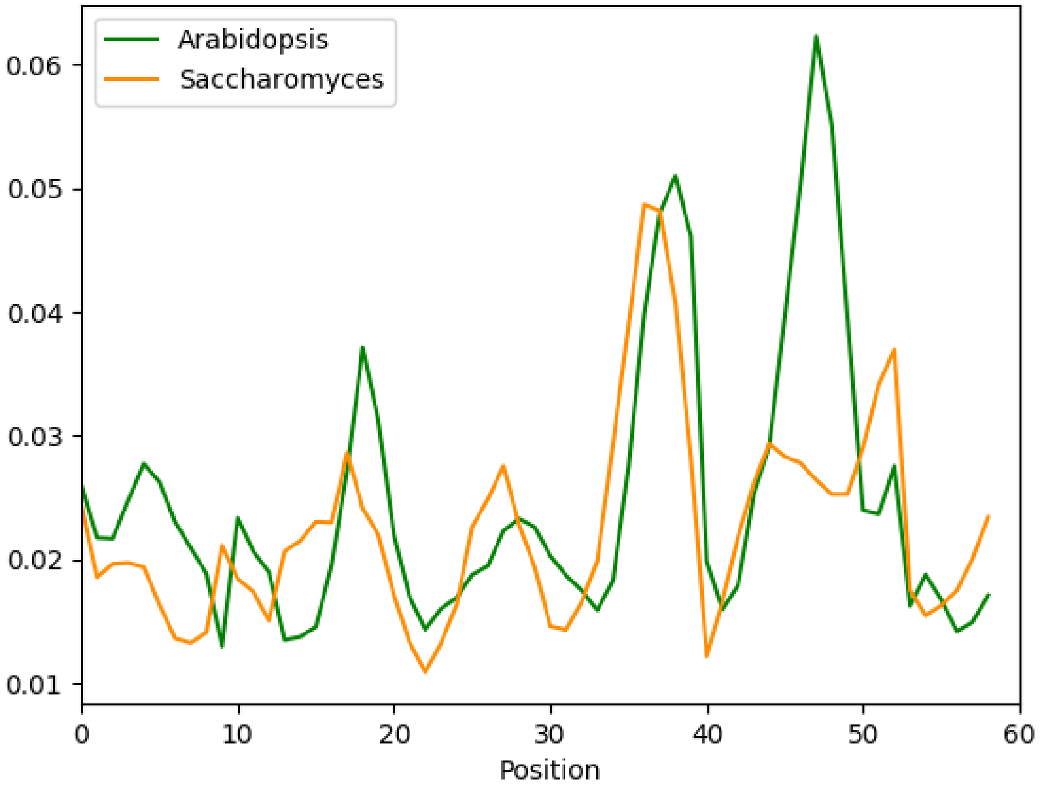

Supplementary Figure 13: **Conservation of RCI in remote homologous proteins.** The RCI values for the oxidized thioredoxin 1 of A. thaliana (green) and S. cervisia (orange). The x-axis represents the aligned position, while the y-axis the RCI value of the two proteins in that specific residue

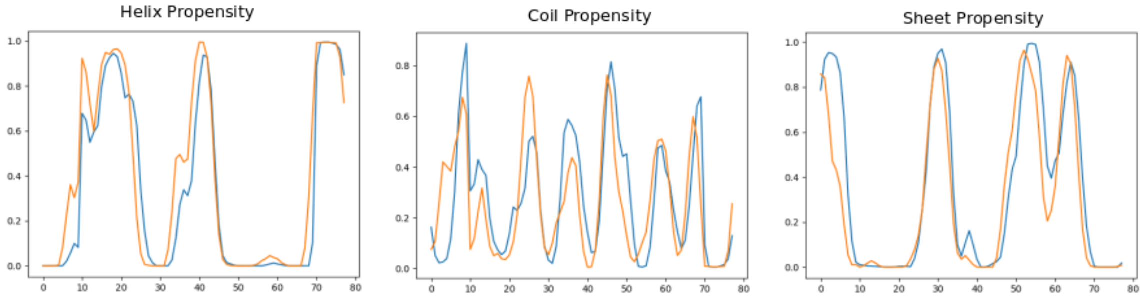

Supplementary Figure 14: **Conservation of d2D in remote homologous proteins.** The secondary structure propensities values determined by d2D for the oxidized thioredoxin 1 of A. thaliana (green) and S. cervisia (orange). The x-axis represents the aligned position, while the y-axis the secondary structure propensity value of the two proteins in that specific residue

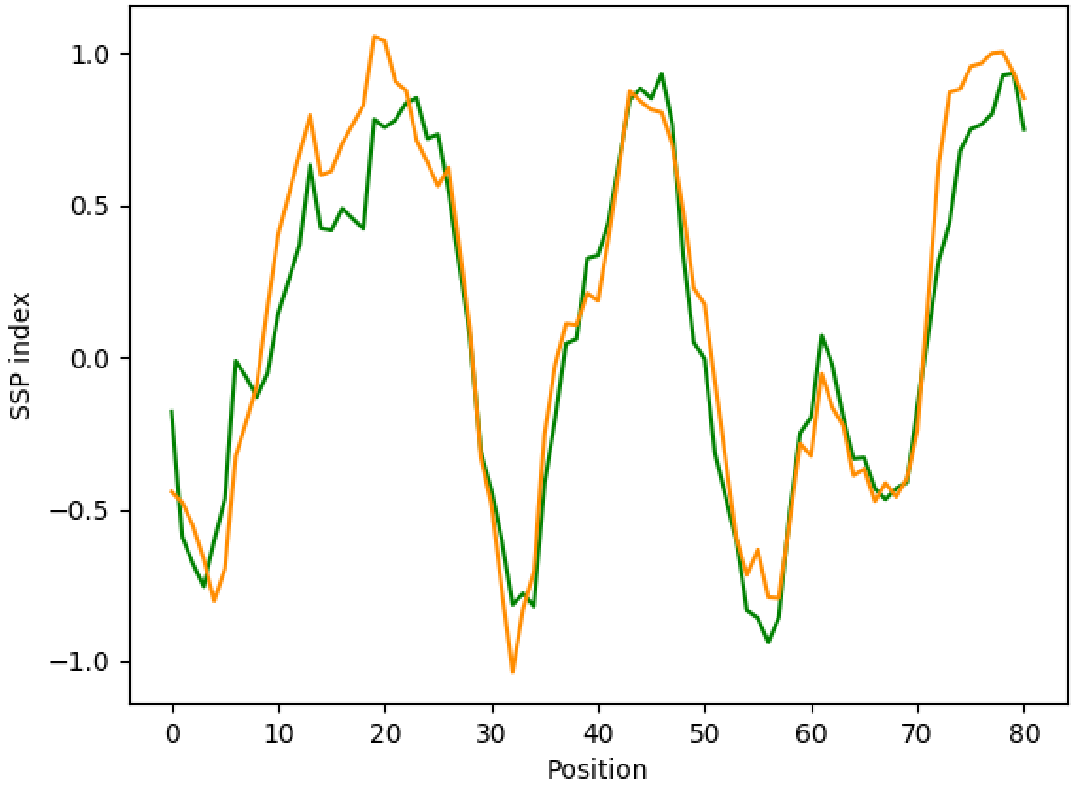

Supplementary Figure 15: **Conservation of SSP in remote homologous proteins.** SSP index for the oxidized thioredoxin 1 of A. thaliana (green) and S. cervisia (orange). The x-axis represents the aligned position, while the y-axis the SSP value of the two proteins in that specific residue

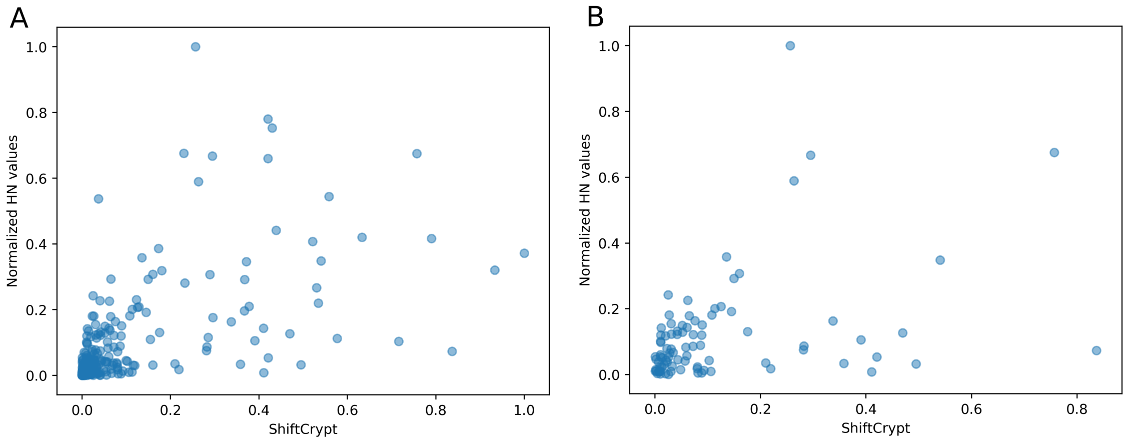

Supplementary Figure 16: **ShiftCrypt and HN changes in monomer/dimer conformations**. Scatter plot between the HN and ShiftCrypt changes in monomer/dimer protein conformations. Plot A shows the correlation between the changes in NH and ShiftCrypt values for all the residues, while plot B takes into consideration only the ones in the interaction patch.

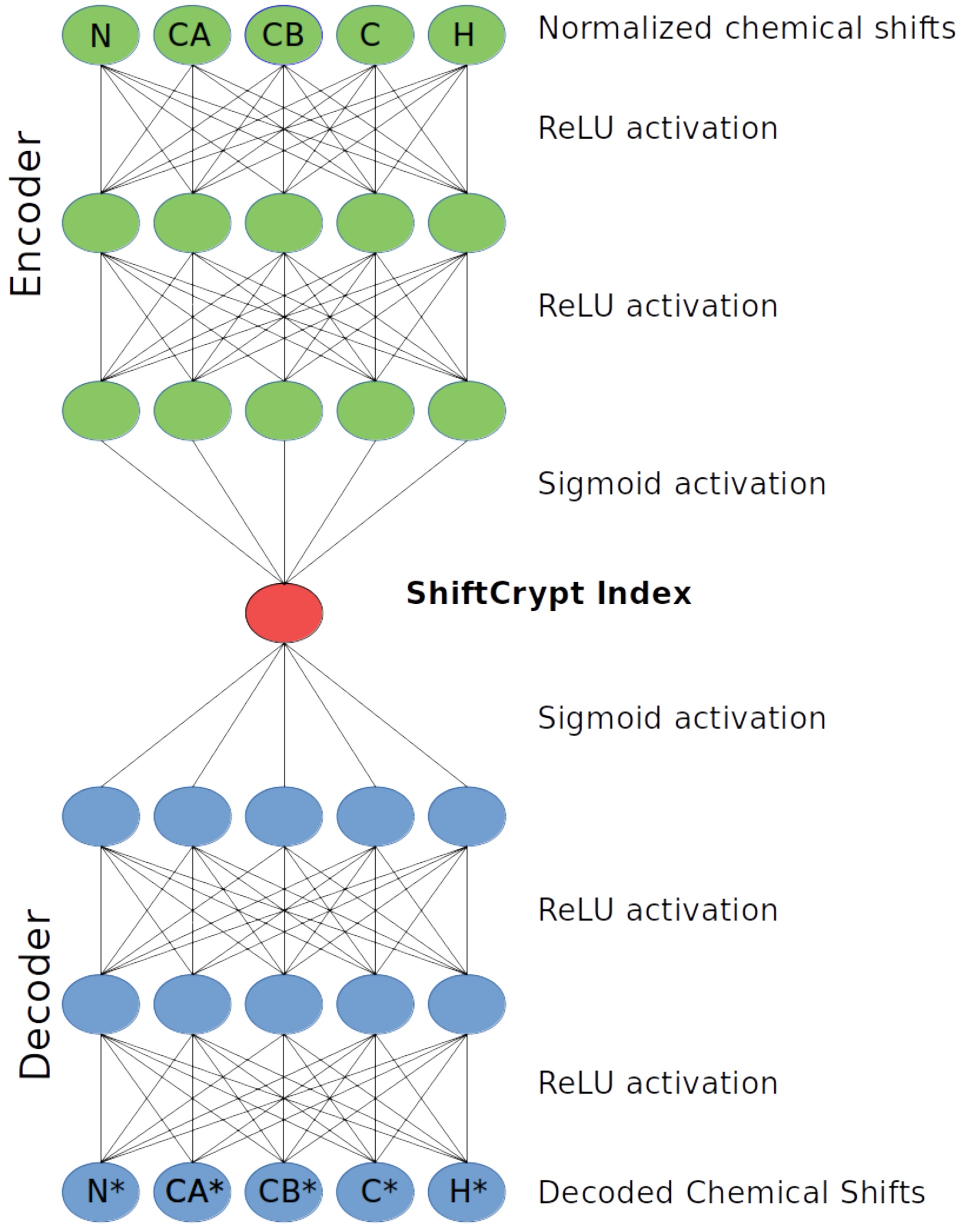

Supplementary Figure 17: **Autoencoder structure.** An overview of the generation of the Shiftcrypt index. The scaled set of chemical shifts for a specific amino acid type is taken as input from the encoder part of the network (green layers). The chemical shift values are then remapped by 3 hidden layers with ReLU activation. The end of the encoding part is a single neuron with sigmoid activation. This neuron (highlighted in red), once the neural network is trained, will generate the ShiftCrypt index. The decoding part (blue layers) takes the said neuron as input and, with a mirrored structure, tries to reproduce the original chemical shift values. In this way the neural network will optimize the information contained in the ShiftCrypt index

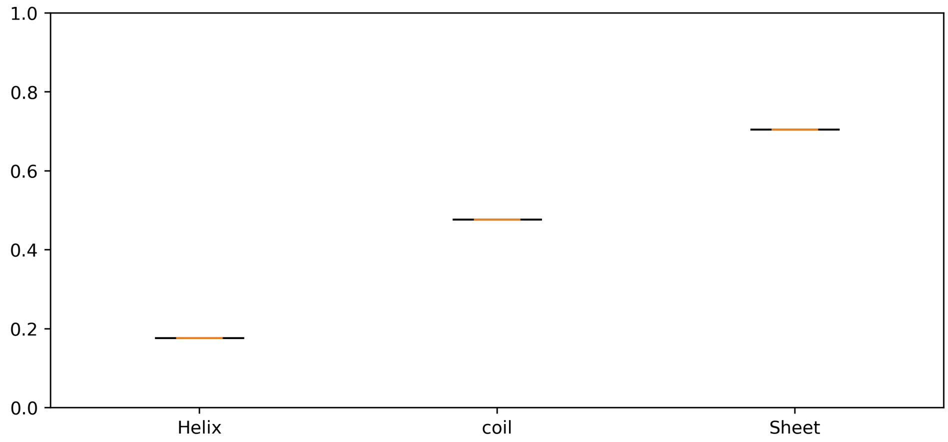

Supplementary Figure 18: **Stability of the model.** The stability of the model varying its parameters. The plot shows the distribution of the medians of the secondary structure populations obtained varying the number of hidden neurons (boxplot with percentiles 25, 50 and 75). The figure shows that different models converge to similar solutions, in which the relationship between ShiftCrypt and secondary structure propensities is maintained.

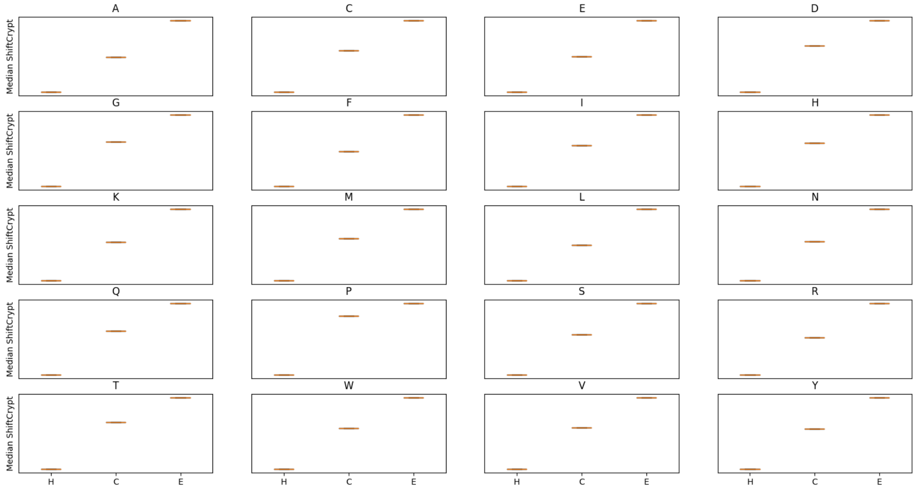

Supplementary Figure 19: **Stability of the model per amino acid type**. The stability of the model varying its parameters and stratifying by amino acid. Similarly to supplementary figure 18, this figure shows that, varying the number of hidden neurons of the model, the relationship between ShiftCrypt and secondary structure propensities is maintained even stratifying by amino acidic type.

Supplementary Tables

Supplementary Table 1: **Secondary structure distributions**. The table shows the value of the integral of the intersection of the distributions shown in Supplementary Figure 2

Residue type	Helix-Coil	Coil-Sheet	Helix-Sheet
A	0.28	0.36	0.12
C	0.46	0.62	0.37
E	0.29	0.37	0.11
D	0.29	0.57	0.21
G	0.36	0.51	0.19
F	0.35	0.47	0.15
I	0.26	0.53	0.12
H	0.41	0.52	0.26
K	0.31	0.47	0.14
M	0.28	0.45	0.13
L	0.27	0.45	0.15
N	0.38	0.5	0.29
Q	0.28	0.45	0.12
P	0.23	0.67	0.1
S	0.33	0.42	0.16
R	0.27	0.41	0.1
T	0.3	0.55	0.18
W	0.36	0.55	0.13
V	0.24	0.54	0.12
Y	0.35	0.45	0.15

Supplementary Table 2: **Secondary structure distributions excluding CA atoms**. The table shows the value of the integral of the intersection of the distributions of the secondary structures excluding CA atoms from the encoding scheme of ShiftCrypt.

Residue type	Helix-Coil	Coil-Sheet	Helix-Sheet
A	0.32	0.35	0.15
C	0.55	0.59	0.37
E	0.29	0.35	0.11
D	0.35	0.55	0.25
G	0.63	0.6	0.28
F	0.42	0.45	0.15
I	0.28	0.54	0.15
H	0.42	0.45	0.19
K	0.34	0.41	0.14
M	0.29	0.43	0.14
L	0.37	0.44	0.17
N	0.4	0.52	0.34
Q	0.29	0.43	0.12
P	0.34	0.85	0.35
S	0.4	0.4	0.2
R	0.31	0.4	0.12
T	0.35	0.49	0.17
W	0.44	0.47	0.15
V	0.26	0.52	0.12
Y	0.4	0.38	0.15

Supplementary Table 3: **Secondary structure distributions excluding CB atoms**. The table shows the value of the integral of the intersection of the distributions of the secondary structures excluding CB atoms from the encoding scheme of ShiftCrypt.

Residue type	Helix-Coil	Coil-Sheet	Helix-Sheet
A	0.28	0.41	0.12
C	0.43	0.54	0.29
E	0.29	0.39	0.12
D	0.29	0.74	0.2
G	0.37	0.53	0.19
F	0.37	0.55	0.17
I	0.26	0.54	0.12
H	0.41	0.53	0.24
K	0.32	0.48	0.15
M	0.29	0.43	0.12
L	0.28	0.48	0.15
N	0.37	0.54	0.3
Q	0.28	0.44	0.12
P	0.24	0.7	0.12
S	0.32	0.4	0.15
R	0.27	0.44	0.09
T	0.28	0.45	0.14
W	0.33	0.61	0.13
V	0.24	0.54	0.11
Y	0.35	0.38	0.12

Supplementary Table 4: **Secondary structure distributions excluding CA and CB atoms**. The table shows the value of the integral of the intersection of the distributions of the secondary structures excluding CA and CB atoms from the encoding scheme of ShiftCrypt.

Residue type	Helix-Coil	Coil-Sheet	Helix-Sheet
A	0.36	0.42	0.19
C	0.59	0.54	0.35
E	0.31	0.39	0.14
D	0.37	0.59	0.25
G	0.65	0.59	0.27
F	0.42	0.53	0.19
I	0.29	0.54	0.14
H	0.45	0.54	0.18
K	0.36	0.49	0.17
M	0.35	0.48	0.14
L	0.43	0.5	0.18
N	0.57	0.62	0.35
Q	0.29	0.44	0.13
P	0.43	0.81	0.41
S	0.5	0.42	0.25
R	0.28	0.56	0.14
T	0.36	0.43	0.18
W	0.42	0.49	0.14
V	0.3	0.51	0.15
Y	0.4	0.48	0.14

Supplementary Table 5: **Encoding scheme of ShiftCrypt**. The atoms included in the encoding scheme of the full atoms model, per amino acid type. Atoms with redundant values are taken just once.

Residue type	Encoding scheme
A	HA, H, N, CA, CB, C, HB*
C	HA, H, N, CA, CB, C, HB2, HB3
E	HA, H, N, CA, CB, C, HB2, HB3
D	HA, H, N, CA, CB, C, HB2, HB3
G	H, N, CA, C, HA2, HA3
F	HA, H, N, CA, CB, C, HB2, HB3
I	HA, H, N, CA, CB, C, HB, HD1*
H	HA, H, N, CA, CB, C, HB2, HB3, HD2
K	HA, H, N, CA, CB, C, HB2, HB3
M	HA, H, CA, CB, C, HB2, HB3
L	HA, H, N, CA, CB, C, HB2, HB3, HD1*, HD2*
N	HA, H, CA, CB, C, HB2, HB3
Q	HA, H, N, CA, CB, C, HB2, HB3
P	HA, CA, CB, CD, C, HB2, HB3, HG2, HG3, HD2, HD3
S	HA, H, N, CA, CB, C, HB2, HB3
R	HA, H, CA, CB, C, HB2, HB3
T	HA, H, N, CA, CB, C, HB, HG2*
W	HA, H, CA, CB, C, HB2, HB3
V	HA, H, N, CA, CB, C, HG1*, HG2*
Y	HA, H, CA, CB, C, HB2, HB3
