# Supplementary material for: Auto-encoding NMR chemical shifts from their native vector space to a residue-level biophysical index
Source: Nat Commun. 2019 Jun 7;10:2511. doi: 10.1038/s41467-019-10322-w (PMC6555786; doi:10.1038/s41467-019-10322-w)
Supplement: Supplementary file 3 — Description of Additional Supplementary Files [file 41467_2019_10322_MOESM3_ESM.pdf]

**Description of Additional Supplementary Files**

**File name**: Supplementary Data 1

**Description**: Pearson's Correlation coefficients between the simplified and actual chemical shifts. For each atom of the encoding scheme (Supplementary Table 5), the file shows the Pearson's Correlation Coefficient between the real chemical shift values and the simplified one, obtained from an encoding-decoding procedure of the autoencoder.
